# On Robustness of Neural Architecture Search Under Label Noise

**DOI:** 10.3389/fdata.2020.00002

**Published:** 2020-02-11

**Authors:** Yi-Wei Chen, Qingquan Song, Xi Liu, P. S. Sastry, Xia Hu

**Affiliations:** ^1^DATALab, Department of Computer Science and Engineering, Texas A&M University, College Station, TX, United States; ^2^Department of Electrical and Computer Engineering, Texas A&M University, College Station, TX, United States; ^3^Department of Electrical Engineering, Indian Institute of Science, Bangalore, India

**Keywords:** deep learning, automated machine learning, neural architecture search, label noise, robust loss function

## Abstract

Neural architecture search (NAS), which aims at automatically seeking proper neural architectures given a specific task, has attracted extensive attention recently in supervised learning applications. In most real-world situations, the class labels provided in the training data would be noisy due to many reasons, such as subjective judgments, inadequate information, and random human errors. Existing work has demonstrated the adverse effects of label noise on the learning of weights of neural networks. These effects could become more critical in NAS since the architectures are not only trained with noisy labels but are also compared based on their performances on noisy validation sets. In this paper, we systematically explore the robustness of NAS under label noise. We show that label noise in the training and/or validation data can lead to various degrees of performance variations. Through empirical experiments, using robust loss functions can mitigate the performance degradation under symmetric label noise as well as under a simple model of class conditional label noise. We also provide a theoretical justification for this. Both empirical and theoretical results provide a strong argument in favor of employing the robust loss function in NAS under high-level noise.

## 1. Introduction

Label noise, which corrupts the labels of training instances, has been widely investigated due to its unavoidability in real-world situations and harmfulness to classifier learning algorithms (Frénay and Verleysen, 2013). Many recent studies have presented both empirical and analytical insights on learning of neural networks under label noise. Specifically, in the context of risk minimization, there are many recent studies on robust loss functions for learning classifiers under label noise (Ghosh et al., 2017; Patrini et al., 2017; Zhang and Sabuncu, 2018).

The neural architecture search (NAS) seeks to learn an appropriate architecture also for a neural network in addition to learning the appropriate weights for the chosen architecture. It has the potential to revolutionize the deployment of neural network classifiers in a variety of applications. One requirement for such learning is a large number of training instances with correct labels. However, generating large sets of labeled instances is often difficult, and the process for labeling (e.g., crowdsourcing) has to contend with many random labeling errors. As mentioned above, label noise can adversely affect the learning of weights of a neural network. For NAS, the problem is compounded because we need to search for architecture as well. Since different architectures are learned using training data and compared based on their validation performance, label noise in training and validation (hold-out) data may cause a wrong assessment of architecture during the search process. Thus label noise can result in undesirable architectures being preferred by the search algorithm, leading to the loss of performance. In this paper, we systematically investigate the effect of label noise on NAS. We show that label noise in the training or validation data can lead to different degrees of performance variation. Recently, some robust loss functions are suggested for learning the weights of a network under label noise (Ghosh et al., 2017; Zhang and Sabuncu, 2018). The standard NAS algorithms use the categorical cross entropy (CCE) loss function. We demonstrate through simulations that the use of a robust loss function (in place of CCE) in NAS can mitigate the effect of harsh label noise. We provide a theoretical justification for this observed performance: for a class of loss functions that satisfies a robustness condition, we show that, under symmetric label noise, the relative risks of different classifiers are the same regardless of whether or not the data are corrupted with label noise.

The main contributions of the paper can be summarized as follows. We provide, for the first time, a systematic investigation of the effects of label noise on NAS. We provide the theoretical and empirical justification for using loss functions that satisfy a robustness criterion. We show that the use of robust loss functions is attractive because of the better performance under high-degree noise than that under the standard CCE loss.

## 2. Background

### 2.1. Robust Risk Minimization

In the context of multi-class classification, the feature vector is represented as $\text{x}\in X\subseteq {\mathbb{R}}^{d}$, and the corresponding class label denotes $y_{\text{x}}\in \left[c\right]=\left\{1\ldots c\right\}=Y$. A classifier $f:X\to {\mathbb{R}}^{c}$ is learned to map each feature vector to a vector of scores, which are later used to decide a class. We assume *f* would be a DNN with the softmax output in this paper. Ideally, we could have a clean labeled dataset $D={\left\{\left({\text{x}}_{i},y_{{\text{x}}_{i}}\right)\right\}}_{i=1}^{n}$ drawn *i.i.d*. from an unknown joint distribution D over (X×Y).

In the presence of label noise, the noisy dataset is represented as $D^{\eta }={\left\{\left({\text{x}}_{i},{ỹ}_{{\text{x}}_{i}}\right)\right\}}_{i=1}^{n}$ sampled *i.i.d*. from the noisy distribution $D^{\eta }$, where ỹ_**x**_ is the noisy label. A noise model could capture the relationship between D and $D^{\eta }$ by:

$$\begin{array}{l} \eta_{\text{x},jk}=Pr\left({ỹ}_{\text{x}}=k|y_{\text{x}}=j,\text{x}\right);\underset{k}{\sum }\eta_{\text{x},jk}=1,\forall j,\text{x}. \end{array}$$

The problem of robust learning of classifiers under label noise can be informally summed up as follows. We get noisy data drawn from $D^{\eta }$ and use it to learn a classifier; however, the learned classifier has to perform well on clean data drawn according to D.

One can consider different label noise models based on what we assume regarding η_**x**,*jk*_ (Frénay and Verleysen, 2013; Manwani and Sastry, 2013; Ghosh et al., 2017; Patrini et al., 2017). In this paper, we consider only symmetric noise and hierarchical (class conditional) noise. If η_**x**,*jk*_ = 1 − η for *j* = *k*, $\eta_{jk}=\frac{\eta }{c-1}$ for *j* ≠ *k*, then the noise is said to be symmetric or uniform. If η_**x**,*jk*_ is a function of (*j, k*) and independent of **x**, then it is called class conditional noise. We consider a particular case where the set of class labels can be partitioned into some subsets, and label noise is symmetric within each subset. We call this hierarchical noise. This is more realistic because, for example, when the labels are obtained through crowdsourcing, it is likely that different breeds of dogs may be confused with each other, although a dog may never be mislabeled as a car.

Here we define the robustness of risk minimization algorithms (Manwani and Sastry, 2013). Given a classifier *f*, its risk under loss function L is defined as $R_{L}={𝔼}_{D}\left[L\left(f\left(\text{x}\right),y_{\text{x}}\right)\right]$ and f^*^ denotes the minimizer of $R_{L}\left(\cdot \right)$. This is often referred to as L-risk to distinguish it from the usual Bayes risk, but we will call it risk here. Similarly, under noisy distribution the risk of *f* is given by $R_{L}^{\eta }\left(f\right)={𝔼}_{D^{\eta }}\left[L\left(f\left(\text{x}\right),{ỹ}_{\text{x}}\right)\right]$ and the corresponding minimizer of $R_{L}^{\eta }\left(\cdot \right)$ is $f_{\eta }^{*}$. We say the loss function L is noise-tolerant or robust if:

$$\begin{array}{l} Pr_{D}\left[Pred◦f^{*}\left(\text{x}\right)=y_{\text{x}}\right]=Pr_{D}\left[Pred◦f_{\eta }^{*}\left(\text{x}\right)=y_{\text{x}}\right], \end{array}$$

where *Pred* ◦ *f*(*x*) denotes the decision on classification scores *f*(*x*) and $Pr_{D}$ denotes probability under the clean data distribution. Essentially, the above equation indicates that the classifiers learned with clean and noisy data both have the same generalization error under the noise-free distribution.

Robustness of risk minimization, as defined above, depends on the specific loss function employed. It has been proved that symmetric loss functions are robust to the symmetric noise (Ghosh et al., 2017; Zhang and Sabuncu, 2018). A loss function L is symmetric if it satisfies Equation 1 (Ghosh et al., 2017).

(1) $$\begin{array}{l} \underset{j}{\sum }L\left(f\left(\text{x}\right),j\right)=C,\forall \text{x},f. \end{array}$$

That is, for any example **x** and classifier *f*, the loss summation over all classes will be equal to a constant *C*. However, the above robustness is defined for finding the minimizer of true risk. One can show that the consistency of empirical risk minimization holds under symmetric noise (Ghosh et al., 2017). Hence, given a sufficient number of examples, empirical risk minimization also would be robust if we use a symmetric loss function.

### 2.2. Robustness of NAS

In this paper, our focus is on NAS. Normally in learning a neural network classifier, one learns only the weights with the architecture chosen beforehand. However, in the context of NAS, one needs to learn both architecture and the weights. Let us denote now by *f* the architecture and by θ the weights of the architecture. Then, the risk minimization can involve two different loss functions as below.

(2) $$\begin{array}{l} f^{*}=\underset{f\in F}{\text{arg min }}{𝔼}_{D_{val}}\left[L_{1}\left(f\left(\text{x};\theta^{*}\right),y_{\text{x}}\right)\right],\\ \theta^{*}=\underset{\theta }{\text{arg min }}{𝔼}_{D_{train}}\left[L_{2}\left(f\left(\text{x};\theta \right),y_{\text{x}}\right)\right]. \end{array}$$

We employ the loss $L_{1}$ for learning architecture while we use $L_{2}$ for learning weights of any specific architecture. Notice from the above that we use the training data to learn the appropriate weights for any given architecture while we use the validation data for learning the best architecture.

The corresponding quantities under the noisy distribution would be:

(3) $$\begin{array}{c} f_{\eta }^{*}=\underset{f_{\eta }\in F}{\text{arg min }}{𝔼}_{D_{val}^{\eta }}\left[L_{1}\left(f_{\eta }\left(\text{x};\theta_{\eta }^{*}\right),{ỹ}_{\text{x}}\right)\right],\\ \theta_{\eta }^{*}=\underset{\theta_{\eta }}{\text{arg min }}{𝔼}_{D_{train}^{\eta }}\left[L_{2}\left(f_{\eta }\left(\text{x};\theta_{\eta }\right),{ỹ}_{\text{x}}\right)\right]. \end{array}$$

For the robustness of NAS, as earlier, we want the final performance to be unaffected by whether or not there is label noise. Thus, we still need that the test error, under noise-free distribution, of f^*^ and $f_{\eta }^{*}$ be the same. However, there are some crucial issues to be noted here.

The parameters θ of each *f* in the search space can be optimized by the empirical risk of $L_{2}$ with *D*_*train*_, and then the best-optimized *f* is selected by the empirical risk of $L_{1}$ with *D*_*val*_. Thus, in NAS, label noise in training data and validation data may have different effects on the final learned classifier. Also, during the architecture search phase, each architecture is trained only for a few epochs, and then we compare the risks of different architectures. Hence, in addition to having the same minimizers of risk under noisy and noise-free distributions, relative risks of any two different classifiers should remain the same irrespective of the label noise.

In NAS, the most common choice for $L_{1}$ is 0–1 loss (i.e., accuracy), while for $L_{2}$ it is categorical cross entropy (CCE). Suppose **p** represents the output of the softmax layer and let the class label of an example be *t*. The CCE is defined by $L\left(\text{p},t\right)=-\text{log}\left(p_{t}\right).$ 0–1 loss is known as symmetric and hence is robust. However, CCE is not symmetric because it does not satisfy Equation 1 (CCE is not bounded). Intuitively, we can mitigate the adverse effects of symmetric noise on NAS by replacing $L_{2}$ with any symmetric loss function. Robust log loss (RLL) (Kumar and Sastry, 2018) is a modification of CCE.

$$\begin{array}{l} L\left(\text{p},t\right)=\log\left(\frac{\alpha +1}{\alpha }\right)-\log\left(\alpha +p_{t}\right)+\overset{c}{\underset{j=1,j\neq t}{\sum }}\frac{1}{c-1}\log\left(\alpha +p_{j}\right) \end{array}$$

where α > 0 is a hyper-parameter and *c* denotes the number of all classes. It satisfies the symmetry condition (Equation 1) and compares (in log scale) probability score of desired output with the average probability score of all other labels. In contrast, the CCE loss only looks at the probability score of the desired output. Another symmetric loss is mean absolute error (MAE) defined by $L\left(\text{p},t\right)=\overset{c}{\underset{j=1}{\sum }}|y_{j}-p_{j}|$. Since MAE takes longer training time to coverage (Zhang and Sabuncu, 2018), we make use of RLL in place of CCE in NAS. For other symmetric loss functions (Charoenphakdee et al., 2019), we leave them for future work.

## 3. Theoretical Result

As discussed earlier, we want a loss function that ensures that the relative risks of two different classifiers remain the same with and without label noise. Here we prove this for symmetric loss functions.

**Theorem 1**. Let L be a symmetric loss function, D be a noise-free distribution, and $D^{\eta }$ be a noisy distribution with symmetric noise $\eta <\frac{c-1}{c}$, where *c* is the number of total classes. The risk of *f* over D is $R_{L}\left(f\right)$, and over $D^{\eta }$ is $R_{L}^{\eta }\left(f\right)$. Then, given any two classifiers *f*_1_ and *f*_2_, if $R_{L}\left(f_{1}\right)<R_{L}\left(f_{2}\right)$, $R_{L}^{\eta }\left(f_{1}\right)<R_{L}^{\eta }\left(f_{2}\right)$ and vice versa.

Proof 1. Though this result is not explicitly available in the literature, it follows easily from the proof of Theorem 1 in Ghosh et al. (2017). For completeness, we present the proof here. For symmetric label noise, we have:^1^

$$\begin{array}{l} R_{L}^{\eta }\left(f\right)={𝔼}_{\text{x},{ỹ}_{\text{x}}}L\left(f\left(\text{x}\right),{ỹ}_{\text{x}}\right)\\ \;={𝔼}_{\text{x}}{𝔼}_{y_{\text{x}}|\text{x}}{𝔼}_{{ỹ}_{\text{x}}|\text{x},y_{\text{x}}}L\left(f\left(\text{x}\right),{ỹ}_{\text{x}}\right)\\ ={𝔼}_{\text{x}}{𝔼}_{y_{\text{x}}|\text{x}}\left[\left(1-\eta \right)L\left(f\left(\text{x}\right),y_{\text{x}}\right)+\frac{\eta }{c-1}\overset{c}{\underset{j\neq y_{\text{x}}}{\sum }}L\left(f\left(\text{x}\right),j\right)\right]\\ \;=\left(1-\eta \right)R_{L}\left(f\right)+\frac{\eta }{c-1}\left(C-R_{L}\left(f\right)\right)\\ \;=\frac{\eta C}{c-1}+\left(1-\frac{\eta c}{c-1}\right)R_{L}\left(f\right). \end{array}$$

Note that *C* is the constant in the symmetry condition (Equation 1), and *c* signifies the number of all classes.

For the third equality, we are calculating expectation of a function of ỹ_**x**_ conditioned on *y*_**x**_ and **x**, where random variable ỹ_**x**_ takes *y*_**x**_ with probability 1 − η and takes all other labels with equal probability.

Thus, $R_{L}^{\eta }\left(f\right)$ is a linear function of $R_{L}\left(f\right)$. Also, since $\eta <\frac{c-1}{c}$, we have $\left(1-\frac{\eta c}{c-1}\right)>0$. Hence, the above shows that $R_{L}\left(f_{1}\right)<R_{L}\left(f_{2}\right)$ implies $R_{L}^{\eta }\left(f_{1}\right)<R_{L}^{\eta }\left(f_{2}\right)$ and vice versa. This completes the proof.

**Remark 1**. Theorem 1 shows that under symmetric loss function, the risk ranking of different neural networks remains the same regardless of noisy or clean data. Since 0–1 loss is symmetric, 0–1 loss as $L_{1}$ in NAS could keep the risk ranking of different neural networks consistent. It indicates that we could discover the same optimal network architecture from noisy validation data as the one from clean validation data theoretically. Besides, f^*^ is proved as the global minimizer for both $R_{L}\left(f\right)$ and $R_{L}^{\eta }\left(f\right)$ if L is symmetric (Ghosh et al., 2017). When we adopt a symmetric loss in $L_{2}$, we can obtain $\theta^{*}=\theta_{\eta }^{*}$. With the above two conditions, as long as $\eta <\frac{c-1}{c}$, $L_{1}$ is 0-1 loss, and $L_{2}$ is symmetric loss, a NAS would be robust to symmetric label noise.

**Remark 2**. Theorem 1 demonstrates that the rank consistency for true risk under noisy and noise-free data. The theorem (Ghosh et al., 2017, Thm.4) points out that the minimization of empirical risk converges uniformly to that of the true risk. With the aid of the theorem, the linear relationship in Theorem 1 would be right as well for empirical risk. This implies that under symmetric loss function, the relative ranking of classifiers for empirical risk (with sufficient samples) would be the same as the true risk under noisy and noise-free data. However, the sample complexity would be higher under noisy labels.

## 4. Experiments

To explore how label noise affects NAS and examine the ranking consistency of symmetric loss functions we designed noisy label settings on CIFAR (Krizhevsky and Hinton, 2009) benchmarks using DARTS (Liu et al., 2019) and ENAS (Pham et al., 2018).

### 4.1. Dataset and Settings

#### 4.1.1. Dataset

The CIFAR-10 and CIFAR-100 (Krizhevsky and Hinton, 2009) consist of 32 × 32 color images with 10 and 100 classes, respectively. Each dataset is split into 45, 000, 5, 000, and 10, 000 as training, validation, and testing sets, following AutoKeras (Jin et al., 2019). All the subsets are preprocessed by per-pixel mean subtraction, random horizontal flip, and 32 × 32 random crops after padding with 4 pixels. We corrupt the training and validation labels by noise and always keep testing labels clean, which is common in literature (Ghosh et al., 2017; Zhang and Sabuncu, 2018). The validation set is used to pick up the best neural architecture during searching and decide the best training epoch during final retraining. Note that the test set is only considered to report the performance.

#### 4.1.2. Noise Construction

We provide theoretical guarantee to the performance of RLL under symmetric noise. Meanwhile, to better illustrate/demonstrate/understand the effectiveness of RLL, we evaluate RLL under both symmetric noisy and hierarchical noise.

- Symmetric noise (Kumar and Sastry, 2018): There is an equal chance that one class is corrupted to be another class. This chance can be captured by a matrix *P*_η_ = η*B* + (1 − η)*I*, whose element in the *i*-th row and *j*-th column is the probability of the true label *i* being changed into label *j*. To be specific, *I* is the identity matrix; all elements of the matrix *B* are $\frac{1}{1-c}$ except that diagonal values are zero, and η is the adjustable noise level. We inject the symmetric noise in CIFAR-10 with η of [0.2, 0.4, 0.6].
- Hierarchical noise (Hendrycks et al., 2018): All label classes can uniformly turn to any other label classes that belong to the same “superclass.” For instance, the “baby” class is allowed to flip to the four different categories (e.g., boy and girl) in the “people” superclass rather than “bed” or “bear”. Since CIFAR-100 inherently provides the superclass information, we add the hierarchical noise into CIFRA-100 with noise level η of [0.2, 0.4, 0.6].

#### 4.1.3. NAS Algorithms

In order to investigate the noisy label problem in NAS, we select representative NAS methods, including DARTS (Liu et al., 2019) and ENAS (Pham et al., 2018). The empirical results on AutoKeras (Jin et al., 2019) could be found in the Supplementary Material as well.

- DARTS searches neural architectures by gradient descent. It assigns different network operations by numeric architectural weights and uses Hessian gradient descent jointly to optimize weights of neural networks and architectural weights. The experiment setting of DARTS can be found in section 1 of the Supplementary Material.
- ENAS discovers neural architectures by reinforcement learning. Although its RNN controller still samples potential network operations by REINFORCE rule (Williams, 1992), ENAS could share the weights of network operations between different search iterations. The experiment setting of ENAS can be found in section 2 of the Supplementary Material.

### 4.2. The Impact of Label Noise on the Performance of NAS

To demonstrate how erroneous labels affect the performance of NAS, we intentionally introduce symmetric noise (η = 0.6) in training labels, validation labels, or both (all noisy). Different NAS methods execute under clean labels (all clean) and these three noisy settings. We evaluate each searcher by measuring the testing accuracy of its best-discovered architecture. Searched networks are retrained with clean labels or polluted labels, denoted as “all clean” and “all noisy,” respectively. The former one shows how noise in the search phase affects the performance of the standard NAS. The latter one reflects how noise alters the search quality of NAS in practical situations. Furthermore, since test accuracy evaluates the search quality, we also include RLL to reduce the noise effect in the retraining phase.

The main results are shown in Table 1. In the clean retraining setting, the optimal network architectures from DARTS and ENAS with noisy labels could result in comparable performance to the ones searched with clean labels. One possible reason is that both DARTS and ENAS adopt the cell search space, which is limited. As long as the networks can be fully retrained by clean labels, they can achieve similar performance. The architectural variance resulting from label noise does not lead to noticeable performance differences. The observation has also been pointed out in Li and Talwalkar (2019).

**Table 1:** NAS on CIFRA-10 with symmetric noise (η = 0.6).

	**DARTS (Liu et al., 2019)**				**ENAS (Pham et al., 2018)**			
	**All clean**	**Noisy valid**	**Noisy train**	**All noisy**	**All clean**	**Noisy valid**	**Noisy train**	**All noisy**
Clean CCE Retrain	96.98	96.22	95.42	96.69	95.84	96.13	95.84	95.88
Noisy CCE Retrain	81.01	78.76	81.35	81.62	79.33	80.46	78.61	80.34
Noisy RLL Retrain	85.63	84.85	87.11	87.53	79.38	80.07	79.22	79.80

*The test accuracy is shown in percentage. Noisy train or noisy valid corrupts training or validation labels, while all noisy pollutes both training and validation labels. NAS algorithms search architectures by CCE under the above settings and retrain the searched architectures by CCE or RLL (α = 0.01)*.

When it comes to retraining the networks with noisy labels, their accuracy drops significantly. The performance differences come from the classical issue of label noise to deep neural networks (Zhang and Sabuncu, 2018). With the help of RLL, we can perceive that the architectures searched by DARTS could achieve better performance, while ENAS does not. Another important observation for ENAS is that the performance under four search settings is comparable. One reason is that the 0-1 loss in ENAS could provide certain robustness to noisy validation labels, which counteracts the negative effect of symmetric noise. Since the search quality of ENAS seems robust to symmetric noise, we do not explore ENAS further in the following experiments.

When we focus on the noisy retraining of DARTS, the performance of “noisy valid” is the lowest one among others. The decrease of search quality is partially because the $L_{1}$ of DARTS is CCE, which is not robust to symmetric loss. DARTS may not be able to rank the performance of different architectures correctly in the setting. The inferior performance from noisy validation labels in other machine learning models has also been proposed in Inouye et al. (2017). Moreover, the “all noisy” searcher is supposed to produce the worst test accuracy since it has both noisy training and validation labels. Surprisingly, the empirical results show that “all noisy” in DARTS even outperforms “all clean.” A possible conjecture is that the “all noisy” searcher is optimized under the same retraining setting, and the resulting network is intentionally designed to adapt to noisy labels. The finding is worthy of conducting further explorations in the future, such as adopting NAS to discover more robust neural architectures. Despite that, we could still find that label noise in the search phase could generally lead to a negative influence on NAS performance. DARTS especially suffers more from noisy validation labels.

### 4.3. Noise Influence of the Risk Ranking

Since NAS aims to find the architectures that outperform others, obtaining a correct performance ranking among different neural networks plays a crucial role in NAS. As long as NAS can recognize the correct performance ranking during the search phase, it should have a high chance to recommend the best neural architecture finally. Theorem 1 reveals that symmetric loss functions have such desired property under symmetric noise situation. To evaluate the practical effects of the theorem, we construct two different neural networks (Table 2) through randomly choosing the network operations as well as the locations of the skip connection. Each network has 8 layers with 36 initial channels. We also exclude the auxiliary layer to avoid its additional loss.

**Table 2:** Two neural network architectures for the ranking of empirical risk.

	**Network architecture 1**	**Network architecture 2**
Normal cell	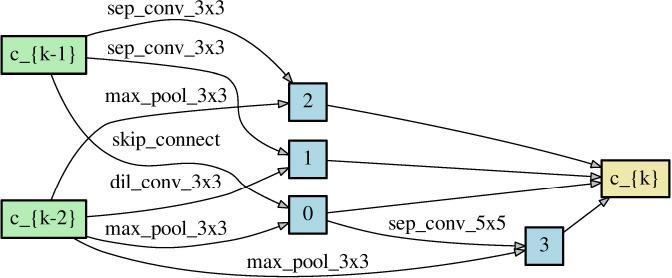	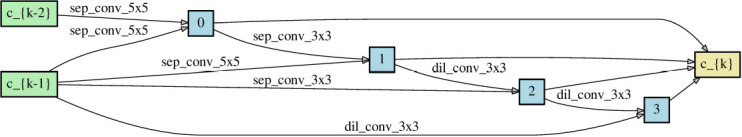
Reduce cell	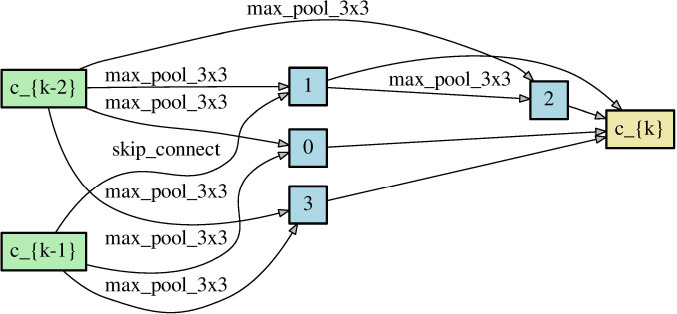	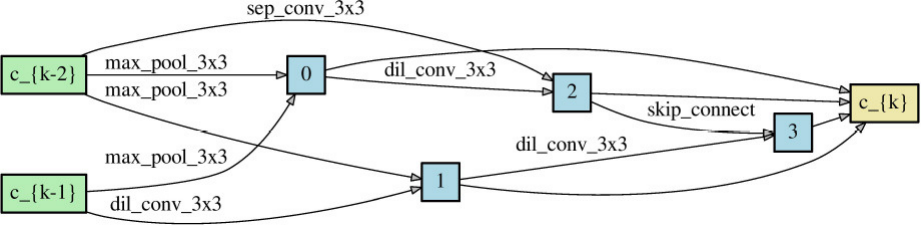

*Each network has eight layers comprising normal cells and reduce cells*.

We train the networks for 350 epochs under clean and noisy training labels, to which symmetric noise of η = 0.6 is injected. Proof 1 of section 3 shows that the noisy true risk is of positive correlation with the clean true risk. Although we do not have the true risk, when the empirical risk of a loss function could conform to the relationship, the loss is supposed to satisfy Theorem 1 likely. Thereby, we inspect the closeness between the empirical noisy risk and its ideal risk, which is computed by the linear function of Proof 1 with the empirical clean risk. To be specific, the Pearson correlation coefficient (PCC) is used to measure the degree of closeness. (0 < *PCC* ⩽ 1 indicates the positive correlation).

**Figure 1:**
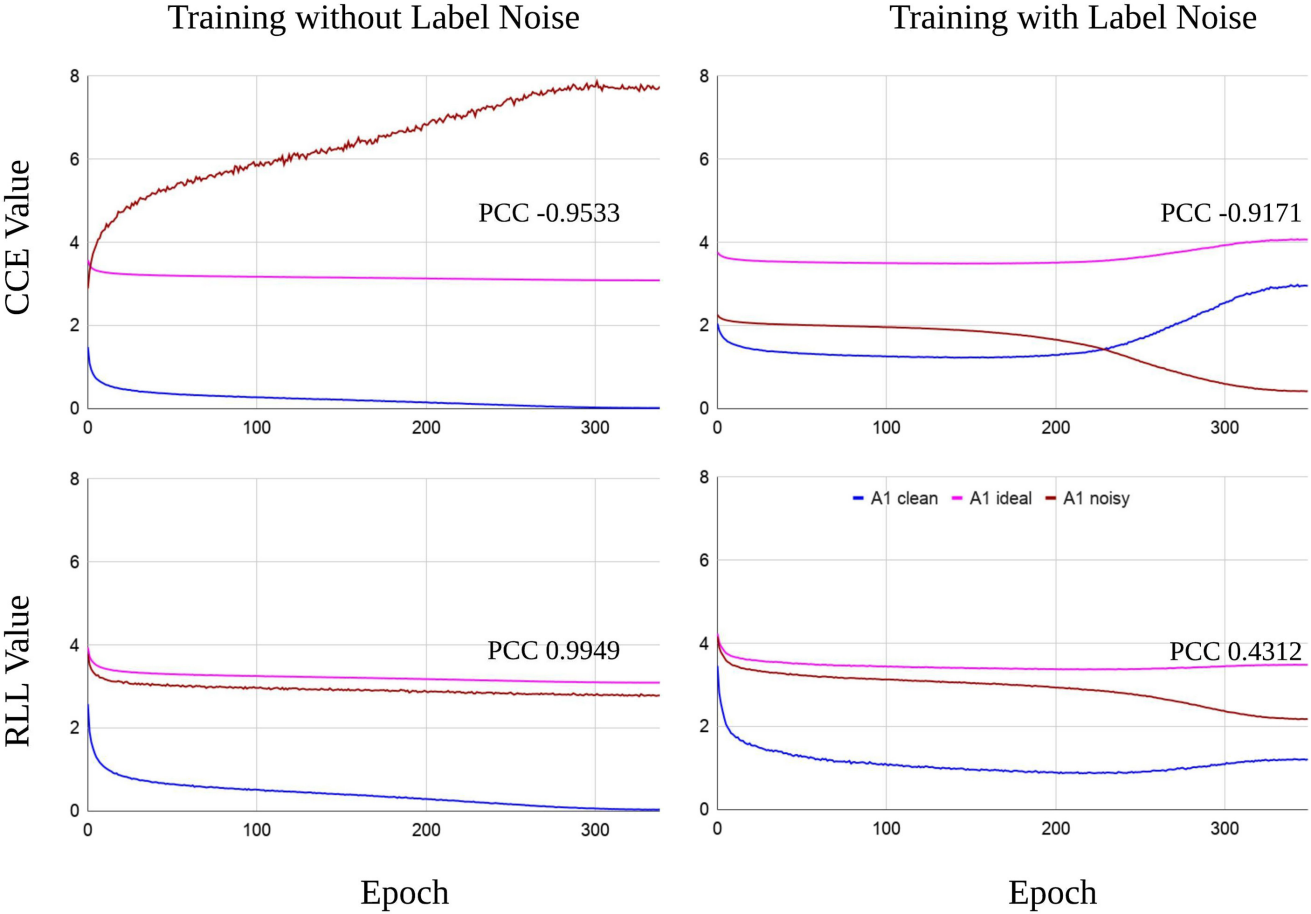
The empirical risk of the first network (depicted in Table 2). The symmetric noise of η = 0.6 is introduced in training labels. The curves of empirical risk (A1 clean and A1 noisy) are from training the network by CCE or RLL (α = 0.01). The ideal curve (A1 ideal) for the noisy risk is computed from Proof 1 of section 3 with A1 clean. When A1 noisy is as close as possible to A1 ideal, the loss could be understood to follow Theorem 1 in practice. As we can see, the bottom RLL figures display that A1 noisy curves are closer to the A1 ideal curves compared to the CCE figures.

Figure 1 displays the RLL and CCE training loss of the first network under noise-free and noisy labels. After we obtained the curve of the empirical clean risk, we drew the ideal curve for the noisy risk according to Proof 1 of section 3. The expectation is that the curve of noisy risk in RLL should be close to the ideal curve, while CCE does not. As we can notice, the curves of noisy risk in CCE deviate from the ideal curves. In contrast, the two curves of noisy risk in RLL stay closer to the ideal curves than CCE. Moreover, the PCC of RLL displays a positive correlation (*PCC* > 0), which also supports that the empirical risk of RLL is very close to the ideal one. The reasons that empirical noisy risks do not perfectly match the ideal one include: (1) training samples (examples) are not enough, (2) hyper-parameters are not optimal for learning the networks, The second network also presents similar results (see Figure S1). Therefore, we could understand that symmetric loss functions have the capability to make the risk ranking under noisy labels uniform to the one under clean labels in practice.

### 4.4. NAS Improvement With Symmetric Loss Function

In practice, the resulting networks from NAS are trained on the potentially wrong labels. We want to see whether NAS could still discover high-performance networks in this harsh environment with the help of symmetric loss function, especially robust log loss (RLL). The performance of neural networks decreases by label noise, but the symmetric loss can alleviate the adverse influence, as shown in Kumar and Sastry (2018). Thus, in the experiment, no matter DARTS searches networks by CCE or RLL, we leverage RLL in the final retrain phase. Apart from DARTS, Resnet-18 He et al. (2016) is also included in the experiment for performance comparison. Moreover, we are interested in how NAS with RLL works in another type of label noise. Here we also report the results beyond the hierarchical noise of CIFAR-100.

The results presented in Table 3 point out that RLL can still help NAS discover high-performance network architectures under high noise levels. No matter in symmetric or hierarchical noise, DARTS with RLL reaches a similar accuracy to DARTS with CCE under η = 0.2 and 0.4, and RLL one outperforms CCE under η = 0.6. One possible reason is that DARTS is robust to mild noise due to its small search space. Nevertheless, severe noise introduces intense uncertainty for DARTS. RLL can help DARTS to determine relatively robust neural architectures in the harsh condition. From the empirical results, we can claim that the symmetric (robust) loss function, RLL, improves the search quality under high-level label noise. More results for another representative searching algorithm, AutoKeras (Jin et al., 2019), can be found in the Supplementary Material.

**Table 3:** NAS with RLL.

	**Symmetric Noise (CIFAR-10)**			**Hierarchical Noise (CIFAR-100)**		
	**0.2**	**0.4**	**0.6**	**0.2**	**0.4**	**0.6**
ResNet-18	92.05 ± 0.40	88.95 ± 0.14	82.77 ± 0.61	61.27 ± 0.60	53.50 ± 0.94	39.99 ± 2.17
DARTS-CCE	**94.91** ± 0.19	**91.02** ± 0.78	83.31 ± 2.88	**67.82** ± 0.70	52.57 ± 1.03	39.22 ± 2.50
DARTS-RLL	94.66 ± 0.67	90.77 ± 1.56	**86.24** ± 0.85	66.47 ± 1.68	**53.68** ± 1.96	**46.41** ± 2.65

*Test accuracy and standard deviation (3 runs) are represented in percentage. DARTS searches architectures with CCE or RLL (α = 0.01), and then the resulting optimal neural network is trained again from scratch by RLL (α = 0.01). Noise contaminates both training and validation labels with different noise levels. Bold font exhibits the best result in each column*.

## 5. Related Work

### 5.1. Neural Architecture Search

Neural architecture search (NAS) is purposed to facilitate the design of network architectures automatically. Currently, the mainstream approaches to achieve NAS include Bayesian optimization (Kandasamy et al., 2018; Jin et al., 2019), reinforcement learning (Zoph and Le, 2017; Cai et al., 2018; Pham et al., 2018; Zoph et al., 2018), evolutionary algorithms (Real et al., 2017, 2019) and gradient-based optimization (Luo et al., 2018; Cai et al., 2019; Liu et al., 2019). Regardless of the different approaches, NAS consists of two phases: the search phase and the final-retrain phase. During the search phase, NAS generates and evaluates a variety of different intermediate network architectures repeatedly. Those networks are trained on the training set for a short time (e.g., tens of epochs). Their performance, measured on the validation set, is used as a guideline to discover better network architectures. In the final-retrain phase, the optimal network architecture will be trained with additional regularization techniques, e.g., Shake-Shake (Gastaldi, 2017), DropPath (Larsson et al., 2017), and Cutout (DeVries and Taylor, 2017). The phase usually takes hundreds of epochs. And then the trained network is evaluated on the unseen test set. In general, the two phases utilize the same training set.

From the perspective of the search space of network architectures, current existing works could be divided into the complete architecture search space (Real et al., 2017; Zoph and Le, 2017; Kandasamy et al., 2018; Jin et al., 2019) and the cell search space (Cai et al., 2018, 2019; Luo et al., 2018; Pham et al., 2018; Zoph et al., 2018; Liu et al., 2019; Real et al., 2019). The first search space allows NAS to look for complete networks and provides a high diversity of resulting network architectures. The second one limits NAS to seek the small architectures for two kinds of cells (normal cell and reduction cell). And it is also required to pre-define the base network architecture to contain the searched cells for evaluation, which implies that many intermediate networks will share similar network architecture. Most existing works usually develop from the cell search space because the size of this search space is significantly smaller than the complete one, and can reduce the enormous search time.

Due to the limited hardware resources, our experiments focus on cell search space, including DARTS (Liu et al., 2019) and ENAS (Pham et al., 2018). We also explore the label noise impact on AutoKeras (Jin et al., 2019). Notice that no similar works have studied the effect of label noise on NAS until we publish the work.

### 5.2. Learning Under Corruption Labels

Great progress has been made in research on the robustness of learning algorithms under corrupted labels (Arpit et al., 2017; Chang et al., 2017; Ghosh et al., 2017; Patrini et al., 2017; Zhang et al., 2017, 2018; Jiang et al., 2018; Ren et al., 2018; Zhang and Sabuncu, 2018). A comprehensive overview of previous studies in this area can be found in Frénay and Verleysen (2013). The proposed approaches for learning under label noise can generally be categorized into a few groups.

The first group comprises mostly label-cleansing methods that aim to correct mislabeled data (Brodley and Friedl, 1999) or adjust the sampling weights of unreliable training instances (Chang et al., 2017; Han et al., 2018; Jiang et al., 2018; Ren et al., 2018; Yu et al., 2019) (adding Co-teaching from Han et al., 2018 and Yu et al., 2019). Another group of approaches treats the true but unknown labels as latent variables and the noisy labels as observed variables so that EM-like algorithms can be used to learn the true label distribution of the dataset (Xiao et al., 2015; Vahdat, 2017; Khetan et al., 2018). The third broad group of approaches aims to learn directly from noisy labels under the generic risk minimization framework and focus on noise-robust algorithms (Manwani and Sastry, 2013; Natarajan et al., 2013; Ghosh et al., 2017; Patrini et al., 2017; Zhang and Sabuncu, 2018). There are two general approaches here. One can construct a new loss function using estimated noise distributions, while the others develop conditions on loss functions so that risk minimization is inherently robust. In either case, they can derive some theoretical guarantees on the robustness of classifier learning algorithms.

All the above approaches are for learning parameters of specific classifiers using data with label noise. In NAS, we need to learn a suitable architecture for the neural network in addition to learning of the weights. Our work differs from the above studies that we discuss the robustness in NAS under corrupted labels, while most of the above works focus on the robustness of training in supervised learning. We investigate the effect of label noise in NAS at multiple levels.

## 6. Conclusion

Neural architecture search is gaining more and more attention in recent years due to its flexibility and the remarkable power of reducing the burden of neural network design. The pervasive existence of label noise in real-world datasets motivates us to investigate the problem of neural architecture search under label noise. Through both theoretical and experimental analyses, we studied the robustness of NAS under label noise. We showed that symmetric label noise adversely the search ability of DARTS, while ENAS is robust to the noise. We further demonstrated the benefits of employing a specific robust loss function in search algorithms. These conclusions provide a strong argument in favor of adopting the symmetric (robust) loss function to guard against high-level label noise. In the future, we could explore that the factors cause DARTS to have superior performance under noisy training and validation labels. We could also investigate other symmetric loss functions for NAS.

## Data Availability Statement

Publicly available datasets were analyzed in this study. This data can be found here: https://www.cs.toronto.edu/~kriz/cifar.html.

## Author Contributions

Y-WC was responsible for the most writing and conducted experiments of DARTS (Liu et al., 2019) and ENAS (Pham et al., 2018). QS proofread the paper and proposed the idea of rank consistency along with PS and XL, and also did the experiments of AutoKeras (Jin et al., 2019) and had the same contribution of Y-WC. XL organized work related to learning under corruption labels. PS wrote the remarks of theoretical results and refined the whole paper. XH supervised the progress and provided helpful discussion.

### Conflict of Interest

The authors declare that the research was conducted in the absence of any commercial or financial relationships that could be construed as a potential conflict of interest.

## Footnotes

^1^Note that expectation of clean data is under the joint distribution of **x**, *y*_**x**_ while that of noise data is under the joint distribution of **x**, ỹ_**x**_.
