# Supplementary figures and images for: On Robustness of Neural Architecture Search Under Label Noise
Source: Front Big Data. 2020 Feb 11;3:2. doi: 10.3389/fdata.2020.00002 (PMC7931895; doi:10.3389/fdata.2020.00002)

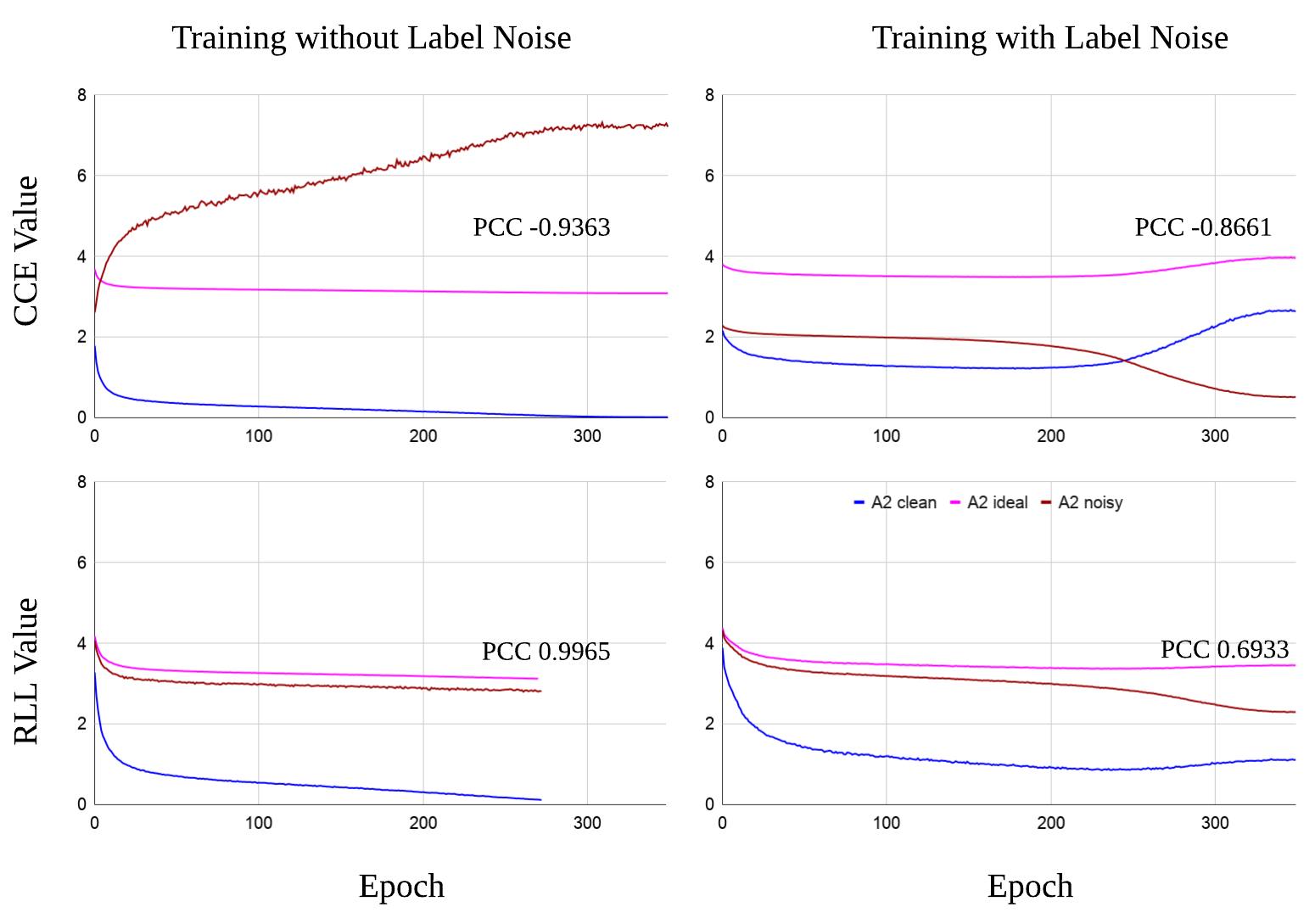

Supplement: Supplementary file 1 [file Image_1.PNG]
