# Supplementary material for: On Robustness of Neural Architecture Search Under Label Noise
Source: Front Big Data. 2020 Feb 11;3:2. doi: 10.3389/fdata.2020.00002 (PMC7931895; doi:10.3389/fdata.2020.00002)
Supplement: Supplementary file 2 [file Data_Sheet_1.PDF]

Supplementary Material

1 EXPERIMENT SETTING OF DARTS

NAS usually has two phases to find the best architecture, search and retrain phase. Here we describe the setting of DARTS for our three experiments. We could search normal and reduce cell architectures for 50 epochs with batch size 64 by using the 8-layer base network with initial channels 16 and dropout path probability 0.3. The optimizer for architectures is Adam with learning rate $3e-4$, L2 penalty $1e-3$, and $(\beta1, \beta2) = (0.5, 0.999)$, while for network parameters is SGD with moment 0.9, L2 penalty $3e-4$, and cosine anneal learning rate initialized by 0.025 and minimal 0.001. For the retraining phase, we take the best cell architectures to generate the 20-layer base network with initial channels 36 and dropout path probability 0.2 and train it for 600 epochs. We also add the auxiliary layer in the network with loss weight 0.4 and use the cutout path 0.4 for image transformation. The optimizer setting is the same as the searching phase except for minimal learning rate 0 and batch size 96. Note that the above configuration is applied to Section 4 if we do not explicitly specify other settings. Since 20-layer base networks in label noise do not perform as good as clean labels, we use the 8-layer base networks for the noisy retraining in Table 1.

2 EXPERIMENT SETTING OF ENAS

We use ENAS to show the negative influence of label noise on the search quality. The experiment setting is as follows. We search normal and reduce cell architectures for 150 epochs with batch size 160 by using the 6-layer base network with initial channels 20 and dropout path probability 0.4. Note that we also add the auxiliary layer with a loss weight of 0.4 in the base network. The optimizer for network parameters is Adam with L2 penalty $1e-4$, $(\beta1, \beta2) = (0.0, 0.999)$, and cosine anneal learning rate initialized by maximal 0.05 and minimal 0.0005. For the controller, we assign the entropy weight $1e-4$, the number of aggregate 10, learning rate 0.0035, tanh constant 1.10, and tanh operation reducing rate 2.5. For the retraining phase, we take the best cell architectures to build the 13-layer base network with initial channels 36 and dropout path probability 0.4 and train it for 630 epochs. The settings of the auxiliary layer and the optimizer are the same as the searching phase except for L2 penalty $2e-4$ and batch size 144.

3 AUTOKERAS WITH SYMMETRIC NOISE

To explore the different NAS algorithms under noisy label settings, we also conduct experiments on AutoKeras Jin et al. (2019). AutoKeras conducts NAS by Bayesian optimization. It designs a neural architecture kernel to compare the similarities among networks and explore new architectures under a tree-based search space with UCB acquisition and Gaussian process estimation. The experimental settings and detailed results are summarized as follows.

3.1 Experimental Setting

We conduct the experiments with the stable version of AutoKeras 0.4 [1]. We conduct a 12-hour search on both CIFAR 10 and CIFAR 100 datasets with four different noise rates and loss combinations of the searching loss and final-fit of the best architecture. The initial architectures are a three-layer convolutional neural network with 64 filters in each layer, DenseNet121, and ResNet-18. The early-stopping strategy is used for both the searching phase and the final-fit phase. All the hyperparameters follow the default settings of the library. The training-testing split ratios are the same as the ENAS and DARTS experiments.

Loss Functions for Search + Final Fit	Symmetric Noise (CIFAR-10)				Hierarchical Noise (CIFAR-100)			
	0.0	0.2	0.4	0.6	0.0	0.2	0.4	0.6
CCE + RLL (0.01)	92.22	88.78	**85.27**	77.29	**69.31**	56.6	50.41	40.55
RLL + RLL (0.01)	**92.51**	**89.21**	85	**77.38**	66.37	**58.41**	**51.01**	**41.04**

Table S1. Results of AutoKeras with different loss combinations for search and final fit. Test accuracy is represented in percentage.

3.2 Experimental Results

We adopt AutoKeras for NAS using either CCE or RLL during the search phase and then refit the resulting optimal neural network from scratch with RLL ($\alpha = 0.01$). From Table S1, two observations could be found. (1) On both symmetric noise and hierarchical noise settings, though using RLL for searching overall outperforms the CCE correspondings, the improvements are quite marginal comparing to the DARTS and ENAS experiments. There are two possible reasons. Firstly, the early stopping strategy partially eliminates the issue by maintaining the rank consistency during the search process. Secondly, comparing to DARTS and ENAS, which take a batch of data to update the searcher (or hyper-graph) during each search epoch, AutoKeras fully trains each network during the search phase, which potentially enhances the ranking consistency and robustness in the NAS process. (2) Though theoretically, we only prove that RLL can provide rank consistency under symmetric noise to some extent, empirically, RLL also provides a certain degree of improvement under the hierarchical noise setting. Analyzing this phenomenon and providing specific theoretical supports for different searching strategies (e.g., Bayesian optimization and reinforcement learning) could be interesting future work.

REFERENCES

Jin, H., Song, Q., and Hu, X. (2019). Auto-keras: An efficient neural architecture search system. *ACM SIGKDD Conference on Knowledge Discovery and Data Mining*

[1] https://autokeras.com/start/

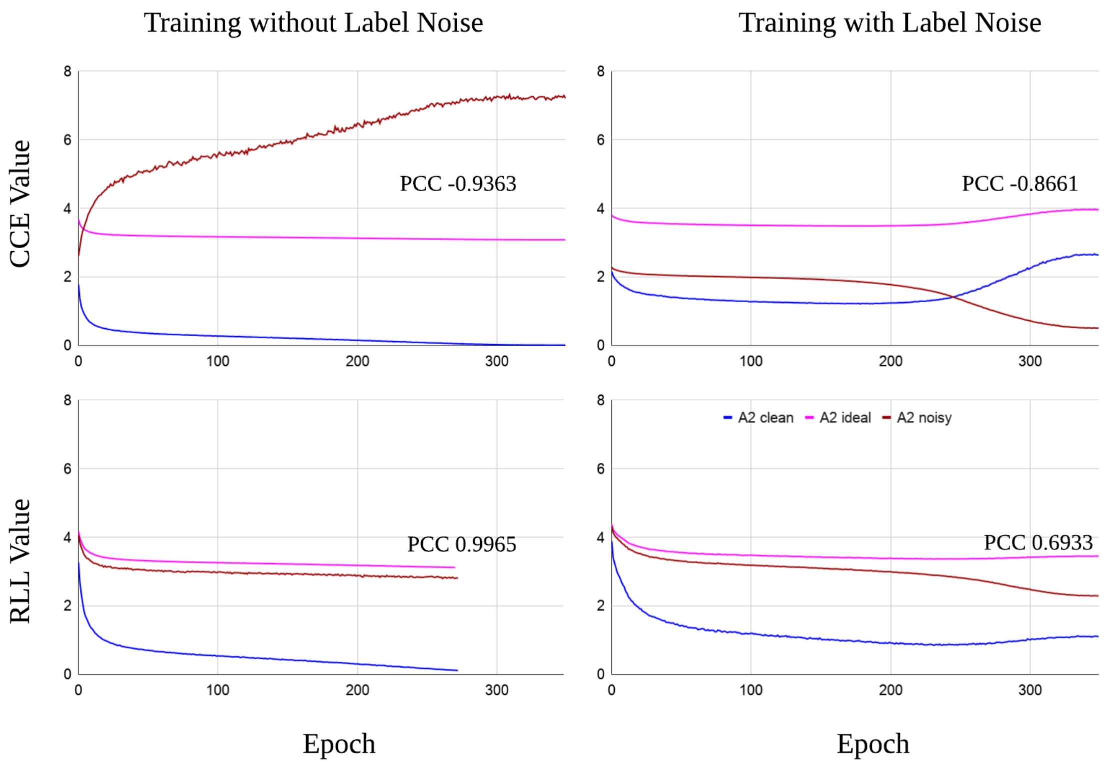

Figure S1. The empirical risk of the second network (depicted in Table 2). The symmetric noise of $\eta = 0.6$ is introduced in training labels. The curves of empirical risk (A2 clean and A2 noisy) are from training the network by CCE or RLL. The ideal curve (A2 ideal) for the noisy risk is computed from Proof 1 of Section 3 with A2 clean. When A2 noisy is as close as possible to A2 ideal, the loss could be understood to follow Theorem 1 in practice. As we can see, the bottom RLL figures display that A2 noisy curves are closer to the A2 ideal curves compared to the CCE figures.
